# Dr. Merryman Drops in

**Published:** 1889-11

**Authors:** 


### DR. MERRYMAN DROPS IN.

"Hello, Daniels!" said the doctor, (he always *will* call us "Daniels") as he dropped lazily into our easy chair, momentarily vacated, and throwing his legs across the desk, rolled and lighted a cigarrette,—"have you seen that "bar" story in one of the late exchanges?"

"No," we replied, "fact is, been too busy to look over the papers received this month; and, besides, I am shoving all that sort of work off on Bennett, you know; he likes it, and good-naturedly, makes abstracts and notes for us; but tell us about it."

"Here it is," said the doctor, "I'll read it. It's headed, 'The Dread of Death;' *should* have been 'Dread-of-being-eaten-alive'; but here it is, in the Medical and Surgical Reporter. I'll read it:"

"Sir Lyon Playfair, in a letter to Junius Henri Browne, author of a paper with the above title, says: "Having represented a large constituency (the University of Edinburgh) for seventeen years as a member of Parliament, I naturally came in contact with the most eminent medical men in Englahd. I have put the question to most of them: 'Did you, in your extensive practice, ever know a patient who was afraid to die?' With two exceptions they answered, 'No.' One of these exceptions was Sir Benjamin Brodie, who said he had seen one case. The other was Sir Robert Christian, who had seen one case, that of a girl of bad character who had a sudden accident. I have known three friends who were partially devoured by wild beasts under apparently hopeless circumstances of escape. The first was Livingstone, the great African traveler, who was knocked on his back by a lion, which begun to munch his arm. He assured me that he felt no fear or pain, and that his only feeling was one of intense curiosity as to which part of the body the lion would take next. The next was Rustem Pasha, now Turkish ambassador in London. A bear attacked him, and tore off part of his hand, and a part of his arm and shoulder. He also assured me that he had neither pain nor fear, but that he felt excessively angry because the bear grunted with so much satisfaction in munching him. The third case is that of Sir Edward Bradford, an Indian officer, now occupying a high position in the Indian office. He was seized in a solitary place by a tiger, which held him firmly behind the shoulder with one paw, and then deliberately devoured the whole of his arm, beginning at the end and ending at the shoulder. He was positive that he had no sensation of fear, and thinks that he felt a little pain when the fangs went through his hand, but is certain that he felt none during the munching of his arm.'—Science, Oct. 18, 1889."

"Now, isn't that a likely yarn?" said the doctor, lighting that everlasting cigarrette, without which he is never seen, "to tell to a lot of doctors; why, what was the fellow doing all the time the bear was a 'chawin' of his elbow?" Why didn't he run,—get further,—or hit the "bar", or choke him off,—or cut him, or shoot 'im? What d'he stand there and let the 'bar' 'chaw' him for? If it 'aint true, it ought to be; and, then, the *lion* part of it? (I had nearly said, the lyin' part of it, which would have been no distinction, as it's all of that sort.) What was *he* doin' *he* didn't get away? And as to his not bein' scared,—great Scott! I'll bet he was as scared as you *looked* when they called on you to respond to the toast 'the ladies.' "

"Did I look scared, doctor?" said we, "Swearingen and Eastland said I got off my 'say' very creditably."

"So you did,—after you got started;—but that lion fellow; I want to know what he was doin', standin' still for a lion to select the softest part of his anatomy, and 'chaw' it at his leisure? And 'not scared, but only consumed by curiosity to know what part the lion would select next!—A pretty story! If I had been tellin' such a yarn, especially to Texans, with any hope of having it believed, I would have had the man, in both instances, to kill the beast;—and 'bar-steak' would have figured in the latter part it;" "but," turning round in our easy chair, and picking up our favorite pen, with which he begun digging viciously at a spot on the desk, "did you see, what Roberts, of the 'Southern Practitioner,' said of the Secretary of the Tennessee State Medical Association, and the volume of Transactions published by him? and how he brings in the Texas Transactions? Did you see that?"

"No; where is it, doctor?"

"Here is Roberts' last," picking it from the pile of late exchanges on the desk, (reading) "The Southern Practitioner, Deering J. Roberts, M. D., editor, Nashville, Tenn., November, 1889. Roberts wields a pointed pen,—if he is on the wrong side of the question of medical legislation to regulate the practice."

"More 'pointed' I hope, Doctor, than the one you are jabbing into the desk there; put it down,—that's a 'private pen.' "

The Doctor threw a look of disgust at us, and evidently thought 'chestnut,' but didn't say it; but continued, after puffing a few times at the inevitable cigarette—his third one,—

"Roberts used to serve on the publishing committee of the Tennessee State Medical Society, and he knowns how Transactions ought to be gotten out; is a good critic. He goes for Dr. Nelson, the secretary, in a way to make one's head swin. Why, he says the most of the many mistakes in the Transactions are in Dr. Nelson's minutes; mistakes,—errors; not typographical errors, but errors of orthography,—bad spelling; and points out for instance, such as—'Tate' for 'Tait,' 'liguor amnia', in twenty places, for 'liquor amnii.' Roberts says it 'makes him sick.'" "He begs the secretary to send out postal cards calling in all the volumes, and then to burn them; and says finally—it is a big load for the society to carry such a secretary—Poor Nelson; Then, oughtn't you fellows to feel good when the New England Monthly says of the Texas Transactions—that the Texas Medical Association,—judging from the 'portly'—yes—'portly' volume (300 pages; do you think that was intended for sarcasm?") —judging from the 'portly' volume before him the Texas State Medical Association must be composed of a lot of intelligent go ahead workers—indefatigable,—or words to that effect; and that the volume of Transactions would be a credit to any State Association."

"But, Doctor, what was it you started out to tell us Roberts said of our Transactions?"

"Why, he rapped Nelson over the nuckles for extravagance in binding the Transactions in cloth; and said the Texas State Medical Association tried binding their Transactions in cloth two years, but abandoned it, and came back to the good old fashioned democratic paper binding of our fathers;—a good joke on Roberts! That paper binding wasn't a matter of preference with your publishing committtee, was it Daniels? Rather because of the insufficiency of the funds to pay for cloth covers, eh?''

''Yes, to tell you the truth Doctor, as a member of the publishing committee and secretary, we got so much praise from the medical press for the Transactions issued in cloth in '85-'86 that it spoilt us, and it went against the grain to have to issue '87-'88 in paper. Speaking individually, I was always rather partial to ''store clothes;'' and if the association had enough money to pay for cloth covers, I for one should never favor paper back Transactions. Our Transactions are worth preserving, and can't be preserved in pamphlet covers; and besides, every member wants a cloth volume. Why, Sir, this year the publishing committee issued 500 copies in cloth—all the treasury could afford, and 150 in paper, and as there are 504 members—why of course, four members, at least, got paper; and we have been kept awake at night, ever since, for fear that next mail would bring four kicks, and complaints at our discriminating in the distribution.''—

'Well?''

''Not to date,'' said we, with a sigh.''—

''Changing the subject'' said the jolly Doctor, ''that 'elixir' business has about subsided, hasn't it? What next? Can't you get up some excitement Daniels?''

''No, I guess not, Doctor. Suppose you try your hand. By the bye, Doctor, I see your subscription has about ex—''[1]

''Ta-ta,'' ''au revoir,'' ''so long,'' said the jolly Doctor, as he leisurely unloaded his legs from the desk, straightened up, yawned, and glided gracefully out at the door, throwing back at us one of his sweetest smiles—''I'll see you later.''

''Call again, Doctor.''

